# AptheraTM Intraocular Lens in a Latino Population: A Case Series

**DOI:** 10.7759/cureus.96197

**Published:** 2025-11-06

**Authors:** Kevin Santiago, Natalio J Izquierdo, José J López-Fontanet, Miguel A Santiago

**Affiliations:** 1 Department of Ophthalmology, School of Medicine, Medical Sciences Campus, University of Puerto Rico, San Juan, PRI; 2 Department of Ophthalmology, Instituto de Ojos de Puerto Rico, Carolina, PRI

**Keywords:** apthera iol, filter ring, hyperfocal, ic-8 apthera lens, small aperture

## Abstract

This study aims to evaluate postoperative vision in Latino patients who underwent the first small-aperture, nontoric, extended depth of focus Intraocular lens implantation (Apthera^TM^) in a Latino population. We conducted a chart review of 49 patients who underwent Apthera^TM^ intraocular lens implantation (Bausch and Lomb, Rochester, NY, USA) in a Latino population. Descriptive analyses and statistical analyses were done.

A total of 49 patients underwent surgery. Of these, there were 18 males (37%) and 31 females (63%). Their ages ranged from 42 to 77 years (mean age = 56.5 years). For the uncorrected distance visual acuity (UDVA, LogMAR) in the Fellow eye (F Eye) and uncorrected near visual acuity (UNVA, LogMAR) in the Apthera™ Eye (Ap Eye), at baseline, the mean preoperative UDVA in the F Eye was 0.255 ± 0.298 LogMAR, with a mean UDVA of 0.068 ± 0.102 LogMAR at one month and 0.059 ± 0.089 LogMAR at six months. In the Ap Eye, the mean UNVA was 0.715 ± 0.372 LogMAR preoperatively, 0.149 ± 0.118 LogMAR at one month, and 0.116 ± 0.154 LogMAR at six months. Mixed-effects regression confirmed that Ap Eyes had significantly higher LogMAR values than F Eyes at both one month (Coef = 0.059, z = 2.09, p* *= 0.037) and six months (Coef = 0.087, z = 2.34, p* *= 0.019).

The mean preoperative spherical equivalent (SE) in the F Eye was +0.353 ± 2.727 D, -0.339 ± 1.852 D at one month, and -0.168 ± 0.541 D at six months. In the Ap Eye, the mean SE was +0.311 ± 2.630 D preoperatively, -1.085 ± 0.885 D at one month, and -1.060 ± 0.861 D at six months. Regression analyses confirmed significantly more myopic outcomes in Ap Eyes compared to F Eyes at both one month (Coef = -0.737,* *z = -3.07,* *p = 0.002) and six months (Coef = -0.91, z = -4.88, p < 0.001).

In the F Eye, the mean astigmatism was -0.609 ± 0.517 D preoperatively, -0.643 ± 0.601 D at one month, and -0.526 ± 0.599 D at six months. In the Ap Eye, the mean astigmatism was -0.781 ± 0.592 D preoperatively, -0.989 ± 0.935 D at one month, and -0.920 ± 0.940 D at six months. Mixed-effects regression suggested a trend toward greater residual astigmatism in Ap Eyes at one month (Coef = -0.307, z = -1.91,p = 0.057) and six months (Coef = -0.35, z = -1.76, p = 0.078), though these did not reach statistical significance.

Our findings show significant improvement in UNVA in the Ap Eye, which is compatible with reports from the IC-8 Apthera^TM ^IOL USFDA clinical study. Regression analyses confirmed significantly more myopic outcomes in Ap Eyes compared to F Eyes at both one month and six months, supporting refractive predictability. In the F Eye, our results are within the expected postoperative goal. Astigmatic errors, however, did not improve in either eye. In conclusion, this surgery benefits both the F Eye and the Ap Eye in Latino patients.

## Introduction

The Apthera^TM^ Intraocular Lens (Ap IOL) (Bausch and Lomb, Rochester, NY, USA) is a novel extended depth-of-focus (EDOF) IOL, based on the previous KAMRA corneal inlay design [1]. This unique IOL uses a small-aperture technology and an embedded FilterRing^TM ^component surrounding it. The FilterRing^TM ^blocks peripheral light rays, which cause blurry vision, and only allows focused central light rays to enter the retina [2]. This is the basis for its EDOF and hyperfocality characteristics, which allow it to correct for presbyopia. EDOF IOL technology creates a single elongated focal point, which provides a continuous range of vision, with excellent distance vision, improved intermediate vision, and functional near vision [3,4]. By achieving mild myopia or near correction of -0.75 spherical diopters in the Ap IOL eye and emmetropia in the fellow eye (F eye), patients will have intermediate and near vision in the Ap IOL eye, and distance vision in the emmetropic F Eye. Further, this lens corrects corneal astigmatism of up to 1.5 astigmatic cylinders [5]. There is no need for axis alignment. This is beneficial compared to toric intraocular lenses, which are designed to decrease astigmatic errors. We report on 49 eyes that underwent cataract surgery with Ap IOL implantation in a Puerto Rican population.

The Ap IOL is a UV-blocking hydrophobic acrylic lens designed to be surgically implanted into the non-dominant or F Eye, as a replacement for a cataractous crystalline lens, which was approved by the FDA in July 2022, based on data from the US Investigational Device Exemption study [2]. The Ap IOL is an aspheric monofocal lens that can filter out peripheral low-optical quality light and deliver high-optical quality light to the retina [6]. The Opaque FilterRing component filters out any peripheral light rays, whereas the small central aperture focuses the entry of only central light rays toward the retina. Due to its distinctive design, it provides a continuous, clear range of vision from near to far. Thus, it works by extending the depth of focus [7,8]. Therefore, the Ap IOL presents an innovative solution for presbyopia.

## Materials and methods

We conducted a chart review of 49 eyes of 49 patients who underwent cataract surgery and Ap IOL implantation by one of the authors (M.A.S.G). Patient identification demographics data included age and gender. Patients’ ophthalmic data included preoperative visual acuities, intraocular pressures, refraction (spherical equivalent), and Ap IOL power.

Inclusion criteria according to the US Investigational Device Exemption study for the Ap IOL are the following: patients of age 22 years-old or older who have been diagnosed with bilateral operable cataracts, who have up to 1.5 D of astigmatic cylinders in the Ap Eye, who do not have a history of retinal disease, and who are not at risk of retinal disease in the future.

Exclusion criteria according to the FDA study are the following: patients whose pupils dilate less than 7.0 mm; patients who have a history of retinal disease, high myopia, diabetes, macular disease, sickle cell disease, retinal tear, retinal detachment, retinal vein occlusion, ocular tumor, and uveitis; and other patients who are predisposed to having retinal disease in the future. 

Ap IOL power calculation was done using Pentacam AXL-wave equipment and the Barrett True K and Barrett Universal II formulas. Barret True K formula is used in eyes that previously underwent refractive corneal surgery, including LASIK, keratotomy, or KAMRA implant. The universal formula is used in patients without previous corneal surgery. The former was used in 10 eyes, and the latter was used in 39 eyes. 

Eleven F Eyes underwent cataract surgery with intraocular implantation with either AcrySof® IQ Monofocal IOL (Alcon) or Clareon® monofocal IOL (Alcon). Twenty-three F Eyes underwent LASIK. Fifteen patients did not have any procedures done in the fellow eye.

In the postoperative period, patients were prescribed ketorolac tromethamine (0.5%) ophthalmic solution three times a day for a month and tobramycin (0.3%) ophthalmic solution four times a day for two weeks. Patients allergic to aspirin were prescribed prednisolone acetate 1% ophthalmic suspension four times a day for a month, instead of ketorolac.

The research followed the tenets of the Declaration of Helsinki, and informed consent was obtained from patients after explaining the nature and possible consequences of the surgeries. The study was approved by the Institutional Review Board of the University of Puerto Rico, Medical Sciences Campus (Protocol Number 2407260677). The authors followed the Health Insurance Portability and Accountability Act (HIPAA) regulations for their patients. Results were collected in MS Excel (Microsoft Corporation, Redmond, Washington, United States). Both descriptive and statistical analyses were done.

## Results

There was a total of 49 patients who underwent surgery. Of these, there were 18 males (37%) and 31 females (63%). Their ages ranged from 42 to 77 years (mean age = 56.5 years). 

Table 1 summarizes UDVA (LogMAR) in the F Eye and UNVA (LogMAR) in the Ap Eye. At baseline, the mean preoperative UDVA in the F Eye was 0.255 ± 0.298 LogMAR, with a mean UDVA of 0.068 ± 0.102 LogMAR at one month and 0.059 ± 0.089 LogMAR at six months. In the Ap Eye, the mean UNVA was 0.715 ± 0.372 LogMAR preoperatively, 0.149 ± 0.118 LogMAR at one month, and 0.116 ± 0.154 LogMAR at six months. Mixed-effects regression confirmed that Ap Eyes had significantly higher LogMAR values than fellow eyes at both one month (Coef = 0.059, z = 2.09, p = 0.037) and six months (Coef = 0.087, z = 2.34, p = 0.019). 

**Table 1:** Preoperative and postoperative uncorrected distance visual acuity in the Fellow eye and uncorrected near vision in the Apthera eye (one-month and six-months follow-up) SD: standard deviation; N: number of patients Coef, z, and p-values were obtained from mixed-effects regression using preoperative values as the reference/baseline

Time point	Eye	N	Mean ± SD	Median	Range (min–max)	Skewness	Kurtosis	Regression Coef	z	p-value
Pre-op	Fellow	49	0.255 ± 0.298	0.18	0-1.3	1.63	5.44	Reference	-	-
	Apthera	49	0.715 ± 0.372	0.70	0-1.3	-0.19	2.22	-	-	-
1 month	Fellow	43	0.068 ± 0.102	0.00	0-0.4	1.60	4.92	Reference	-	-
	Apthera	36	0.149 ± 0.118	0.13	0-0.6	1.54	7.09	0.059	2.09	0.037
6 months	Fellow	34	0.059 ± 0.089	0.00	0-0.3	1.36	3.95	Reference	—	—
	Apthera	31	0.116 ± 0.154	0.10	0-0.54	1.54	4.61	0.087	2.34	0.019

As shown in Table 2, the mean preoperative spherical equivalent (SE) in the F Eye was +0.353 ± 2.727 D, -0.339 ± 1.852 D at one month, and -0.168 ± 0.541 D at six months. In the Ap Eye, the mean SE was +0.311 ± 2.630 D preoperatively, -1.085 ± 0.885 D at one month, and -1.060 ± 0.861 D at six months. Regression analyses confirmed significantly more myopic outcomes in Ap Eyes compared to F Eyes at both one month (Coef = -0.737, z = -3.07, p = 0.002) and six months (Coef = -0.91, z = -4.88, p < 0.001).

**Table 2:** Preoperative and postoperative spherical equivalents in the Fellow and Apthera Eyes SD: standard deviation; N: number of patients Coef, z, and p-values were obtained from mixed-effects regression using preoperative values as the reference/baseline

Time point	Eye	N	Mean ± SD	Median	Range (min–max)	Skewness	Kurtosis	Regression Coef	z	p-value
Preop	Fellow	49	0.353 ± 2.727	0.75	-13-4.75	-2.84	13.75	Reference	-	-
	Apthera	49	0.311 ± 2.630	0.75	-11-4.5	-2.16	9.03	-	-	-
1 month	Fellow	48	-0.339 ± 1.852	-0.125	-12-2.25	-5.26	34.30	Reference	-	-
	Apthera	47	-1.085 ± 0.885	-1.00	-3.25-1.25	0.01	3.91	-0.737	-3.07	0.002
6 months	Fellow	29	-0.168 ± 0.541	-0.25	-1.75-1	-0.42	4.29	Reference	-	-
	Apthera	29	-1.060 ± 0.861	-1.00	-3-1.25	0.17	4.24	-0.910	-4.88	<0.001

Table 3 shows that in the F Eye, the mean astigmatism was -0.609 ± 0.517 D preoperatively, -0.643 ± 0.601 D at one month, and -0.526 ± 0.599 D at six months. In the Ap Eye, the mean astigmatism was -0.781 ± 0.592 D preoperatively, -0.989 ± 0.935 D at one month, and -0.920 ± 0.940 D at six months. Mixed-effects regression suggested a trend toward greater residual astigmatism in Ap Eyes at one month (Coef = -0.307, z = -1.91, p = 0.057) and six months (Coef = -0.35, z = -1.76, p = 0.078).

**Table 3:** Astigmatic errors in the preoperative and postoperative periods SD: standard deviation; N: number of patients Coef, z, and p-values were obtained from mixed-effects regression using preoperative values as the reference/baseline

Time point	Eye	N	Mean ± SD	Median	Range (min–max)	Skewness	Kurtosis	Regression coef	z	p-value
Pre-op	Fellow	49	-0.609 ± 0.517	-0.50	-2-0	-0.94	3.32	Reference	-	-
	Apthera	49	-0.781 ± 0.592	-0.75	-2.5-0	-0.65	3.14	-	-	-
1 month	Fellow	46	-0.643 ± 0.601	-0.50	-3-0	-1.65	6.73	Reference	-	-
	Apthera	47	-0.989 ± 0.935	-0.75	-4.75-0	-1.89	7.55	-0.307	-1.91	0.057
6 months	Fellow	29	-0.526 ± 0.599	-0.50	-3.25-0	-3.29	15.99	Reference	-	-
	Apthera	28	-0.920 ± 0.940	-0.75	-4.5-0	-2.37	9.07	-0.347	-1.76	0.078

Postoperative patients’ complaints included photophobia, positive dysphotopsia, blurred vision, posterior capsule opacification (PCO), and difficulty driving at night. Four patients underwent YAG capsulotomy in the Ap Eye. In our study, there was no endophthalmitis. 

## Discussion

Uncorrected visual acuity

Although the IC-8/Apthera PMA (SSED) used a non-inferiority margin of 0.10 logMAR and set clinical success criteria that included ≥50% of subjects achieving 0.10 logMAR or better [2], the measured mean monocular UDVA in the F Eyes at six months in the FDA study was 0.034 logMAR (SD 0.126) [2]. In our study, mean UDVA in the F Eye improved from 0.255 ± 0.298 logMAR preoperatively to 0.068 ± 0.102 at one month and 0.059 ± 0.089 at six months. Our findings were consistent with distance vision outcomes reported in the FDA trial outcomes.

Previous reports describe substantial improvements in near vision with small-aperture lenses [4,7-11]. The FDA SSED reports mean monocular UNVA for IC-8 eyes of 0.206 logMAR (SD 0.157) at six months [2]. In our study, the Apthera™ eye improved from 0.715 ± 0.372 LogMAR preoperatively to 0.149 ± 0.118 at one month and 0.116 ± 0.154 at six months, surpassing the mean UNVA reported in the SSED [2] and supporting the near-vision advantage of the Apthera™ design, as also noted by Dick et al. [7] and Hooshmand et al. [11]. Mixed-effects regression confirmed that Ap Eyes had significantly higher LogMAR values than fellow eyes at both one month (Coef = 0.059, z = 2.09, p = 0.037) and six months (Coef = 0.087, z = 2.34, p = 0.019). This reflects the expected tradeoff between near gain and slight fellow/Apthera distance differences.

Refractive error values

The FDA labeling and SSED note that the intended refractive target for the Ap Eye is −0.75 D, while the F Eye is expected to be targeted toward emmetropia (plano to −0.50 D MRSE) [2]. In our study, the mean preoperative SE in the F Eye was +0.353 ± 2.727 D. At one month and six months postoperatively, the F Eye shifted to a slight myopic outcome (-0.339 ± 1.852 D at one month, -0.168 ± 0.541 D at six months). This reflects the surgeon’s preference (M.A.S.G) for mild myopia to avoid anisometropia.

Preoperatively, the mean SE in Ap Eyes was +0.311 ± 2.630 D in our study. Our patients then achieved a mean SE of -1.085 ± 0.885 D at one month and -1.060 ± 0.861 D at six months. Regression analyses confirmed significantly more myopic outcomes in Ap Eyes compared to F Eyes at both one month (Coef = -0.737,* *z = -3.07,* *p= 0.002) and six months (Coef = -0.91, z = -4.88,** **p< 0.001), consistent with the intended mini-monovision strategy and the FDA clinical data [2].

Astigmatism

Ang has shown that small-aperture optics maintain useful distance acuity through induced cylinder defocus up to approximately 1.5 D [5,12], supporting the device’s labeled indication for up to 1.5 D preoperative corneal astigmatism [2,4]. In our patients, the mean preoperative astigmatic error in the Ap Eye was -0.781 ± 0.592 D. Postoperatively, the Ap Eyes demonstrated slightly higher residual astigmatism (-0.989 ± 0.935 D at one month, -0.920 ± 0.940 D at six months) compared to F Eyes. Mixed-effects regression suggested a trend toward greater residual astigmatism in Ap Eyes at one month (Coef = -0.307, z = -1.91,p = 0.057) and six months (Coef = -0.35, z = -1.76, p = 0.078), though these did not reach statistical significance. Clinically, the small-aperture mechanism is expected to allow functional vision despite modest residual cylinder, in agreement with prior reports [4,6-9,13].

Limitations

Limitations of our study include the small number of patients who underwent surgery and that some patients were lost to follow-up and did not show up for the one-month and/or six-month postoperative visit.

Future directions

Prospective, larger studies in Latino populations are warranted to confirm long-term stability, refractive predictability, and evaluate for potential complications in patients who receive Apthera™ IOL implantation.

## Conclusions

In summary, our findings show a drastic improvement in UNVA in the Ap Eye, which is compatible with reports from the IC-8 Apthera^TM ^IOL USFDA clinical study. Our results matched the mean uncorrected near visual acuity of 0.206 LogMAR reported at six months in the FDA study. In our study, mixed-effects regression confirmed that Ap Eyes had statistically significantly higher LogMAR values than fellow eyes at both one month and six months. After just one month, the mean near UNVA was better than expected.

Regression analyses confirmed significantly more myopic outcomes in Ap Eyes compared to F Eyes at both one month and six months, supporting refractive predictability. In the F Eye, our results are within the expected postoperative goal. Astigmatic errors, however, did not improve in either eye. In conclusion, this surgery benefits both the F Eye and the Ap Eye in Latino patients. 
